# Digital PCR can provide improved *BCR-ABL1* detection in chronic myeloid leukemia patients in deep molecular response and sensitivity of standard quantitative methods using EAC assays

**DOI:** 10.1016/j.plabm.2021.e00210

**Published:** 2021-03-09

**Authors:** Dagmar Smitalova, Dana Dvorakova, Zdenek Racil, Marianna Romzova

**Affiliations:** a Department of Molecular Medicine, Central European Institute of Technology, Masaryk University, Brno, Czech Republic; b Department of Internal Medicine, Hematology and Oncology, Faculty of Medicine, Masaryk University, Brno, Czech Republic; c Centre of Molecular Biology and Gene Therapy, University Hospital Brno, Czech Republic; d Internal Haematology and Oncology Clinic, University Hospital Brno, Czech Republic; e Institute of Hematology and Blood Transfusion, Prague, Czech Republic

**Keywords:** Chronic myeloid leukemia, *BCR-ABL1* monitoring, GeneXpert BCR-ABL Monitor assay, RT-qPCR, Digital PCR, qPCR, quantitative PCR, RT-qPCR, reverse-transcription quantitative PCR, CML, chronic myeloid leukemia, dPCR, digital PCR, MR, molecular response, TKI, tyrosine kinase inhibitors, IS, international scale, FPR, false positivity rate, EAC, Europe Against Cancer, pDNA, plasmid DNA, LOB, limit of blank, LOD, limit of detection, cDNA, complementary DNA, NTC, no template control

## Abstract

*BCR-ABL1* molecular detection using quantitative PCR (qPCR) methods is the golden standard of chronic myeloid leukemia (CML) monitoring. However, due to variable sensitivity of qPCR assays across laboratories, alternative methods are tested. Digital PCR (dPCR) has been suggested as a robust and reproducible option. Here we present a comparison of droplet dPCR with routinely used reverse-transcription qPCR (RT-qPCR) and automated GeneXpert systems. Detection limit of dPCR was above 3 BCR*-ABL1* copies, although due to background amplification the resulting sensitivity was 0.01% *BCR-ABL1* (MR4.0). Nevertheless, in comparison with GeneXpert, dPCR categorized more than 50% of the patients into different MR groups, showing a potential for improved *BCR-ABL1* detection.

## Highlights

• dPCR assays attain MR4.0 sensitivity due to a blank amplification 3 BCR*-ABL1* copies.
• Quantification principle of RT-qPCR and dPCR introduces bias in copy numbers.
• dPCR provides more sensitive *BCR-ABL1* measurement in >50% MR3.0-MR4.5 CML patients.

## 1 Introduction

Specific targeting of the Bcr-Abl1 enzyme by tyrosine kinase inhibitors (TKI) revolutionized the management of chronic myeloid leukemia (CML) to the point that TKIs can offer near normal life expectancy for CML patients [1]. However, some CML patients do not achieve optimal response at defined treatment time points, and others even develop TKI resistance. Therefore, molecular monitoring is crucial for clinical management of CML [2]. Currently, quantitative reverse-transcription PCR (RT-qPCR) is the technique of choice used to assess *BCR-ABL1* transcript levels in the clinics. However, it often shows insufficient sensitivity and inconsistent detection of minimal *BCR-ABL1* levels [3]. Despite standardization of RT-qPCR results relative to an international scale (IS), issues with reproducibility across laboratories still persist [3,4].

Digital PCR (dPCR) offers high reproducibility, precision and increased sensitivity for rare target detection [5]. Recent studies evaluating the suitability of dPCR as an alternative technique for *BCR-ABL1* monitoring suggest that dPCR has increased sensitivity up to MR5.5, although higher false positivity rate (FPR) could lower the detection limit of this method [[6], [7], [8], [9], [10]]. Therefore, whether dPCR is indeed suitable for monitoring of CML patients remains unclear.

The objectives of our study were to evaluate the performance of droplet dPCR using the Europe Against Cancer (EAC) standardized *BCR-ABL1* and *ABL1* assays and to evaluate the suitability of dPCR for molecular monitoring of CML patients with low or undetectable levels of *BCR-ABL1* in comparison with routinely used methods.

## 2 Materials and methods

### 2.1 Samples

A retrospective analysis was performed on 70 clinical samples from chronic phase CML patients and 15 samples from healthy volunteers used as *BCR-ABL1* negative controls. All samples were obtained from the Department of Internal Medicine, Haematology and Oncology, University Hospital Brno. Characteristics of CML patients are listed in Supplementary Table 1. Informed consent was obtained from all enrolled subjects prior to participation in the study. The study protocol and subject sampling were reviewed and approved by an institutional ethical committee in agreement with the Declaration of Helsinki. Sample handling and storage is described in detail in Supplementary Data.

### 2.2 Calibrators

ERM AD623 BCR-ABL pDNA (Sigma-Aldrich) plasmid set [11] was used to assess the linearity of EAC assays by dPCR, according to the manufacturer’s recommendations. A Qiagen Ipsogen BCR-ABL1 Mbcr Fusion Gene Standard kit and an Ipsogen ABL Control Gene 3 Standard kit were used for quantification of *BCR-ABL1* transcript by RT-qPCR in accordance with the manufacturer’s recommendations.

### 2.3 Quantitative PCR

All qPCR measurements were performed at the Centre of Molecular Biology and Gene Therapy, University Hospital Brno. Two methods, routinely performed to assess treatment response, were used: conventional RT-qPCR and the GeneXpert-based assay.

RT-qPCR quantification of *BCR-ABL1* in K562 ​cells was performed on an Applied Biosystems 7300 Real-Time PCR System. Cepheid Xpert BCR-ABL Monitor test was used for quantification of *BCR-ABL1* in clinical CML samples, according to the manufacturer’s recommendations. Reverse transcription and qPCR reaction conditions are described in detail in Supplementary Data and Table 1.

**Table 1:** Summary of qPCR and dPCR workflow details.

	Quantitative RT-PCR		Digital PCR
	GeneXpert IV	ABI 7300	QX200
**RT**	CML cartridge	SSII∗/VILO	SSII∗/VILO
**cDNA dilution**	CML cartridge	1:2∗/2:1	1:2∗/2:1
**RNA/PCR reaction**	200 ​μL of blood	~200 ​ng∗/~135 ​ng	~200 ​ng∗/~135 ​ng
**Reaction/sample volume**	CML cartridge	40 μL/5μL	40 μL/5μL
**Pre-PCR processing**	No	No	Droplet generation
**Standard**	No	Yes	No
**PCR type**	Nested RT-PCR	Real-Time PCR	End point PCR
**Assay reaction**	Multiplex	Singleplex	Singleplex
**Results**	Cq values	Relative copy numbers	Absolute copy numbers
	ΔΔCq	Copies/sample	Copies/sample
**Analysis**	% ratio (IS)	% ratio x CF (IS)	% ratio

SSII - SuperScript II; VILO SuperScript VILO; Cq - cycle quantification; CF- conversion factor; IS - International scale; ∗conditions used for comparison of dPCR with RT-qPCR.

### 2.4 Digital PCR

All dPCR measurements were performed at the Central European Institute of Technology of Masaryk University Brno, Department of Molecular Medicine on Bio-Rad QX200 Droplet Digital PCR System (dPCR) according to the manufacturer’s recommendations. Reaction conditions are described in Supplementary Data and Table 1.

### 2.5 Statistical analysis

The formulae for calculation of FPR, limit of blank (LOB) and limit of detection (LOD) are concluded in Supplementary Data. Statistical analyses were generated using GraphPad Prism v.9 software. P values ​< ​0.05 were considered significant.

## 3 Results

### 3.1 Detection limit of dPCR using EAC assays

Evaluation of EAC assays’ linearity on dPCR showed near perfect correlation of expected and measured *BCR-ABL1* and *ABL1* copies across the dilution range of the ERM AD623 standard down to 10 copies, with R^2^_*BCR-ABL1*_ ​= ​0.998 and R^2^_*ABL1*_ ​= ​0.999, respectively (Supplementary Fig. 1).

Assessment of FPR was performed on a total number of 80 negative samples, including no template controls (NTC), and the samples from *BCR-ABL1* negative controls. The FPR of NTC (n ​= ​28) was 4% with LOB ​= ​1.4 copies/sample, and the FPR of *BCR-ABL1* negative controls (n ​= ​52; 15 samples tested in 3 and more replicates) was 6% with LOB ​= ​3.2 copies/sample (Supplementary Table 2). A detection limit (LOD) of dPCR, calculated from negative controls, was 3.3 copies/sample (corresponding to 2 positive droplets).

**Fig. 1:**
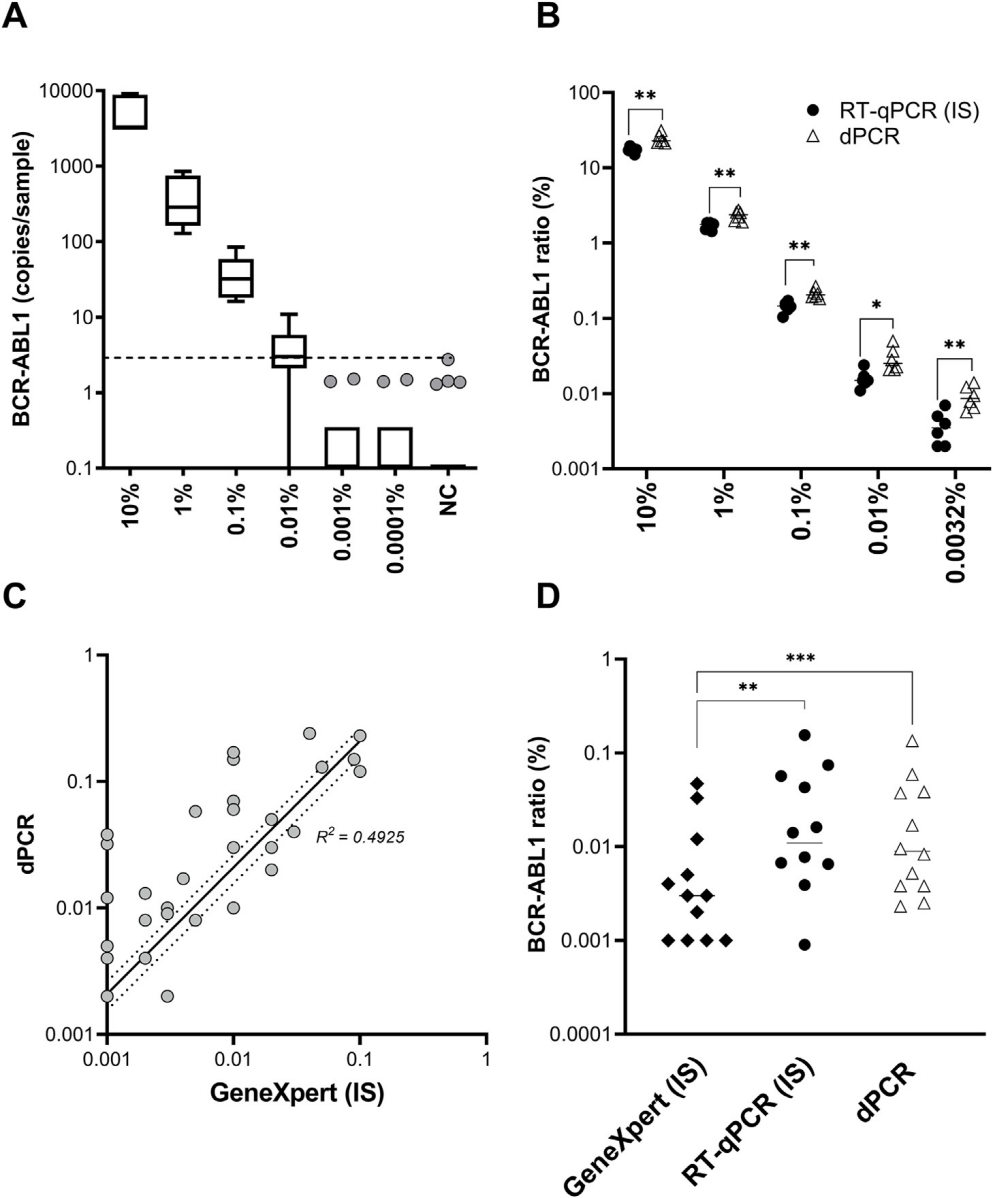
Sensitivity and performance of *BCR-ABL1* and *ABL1* EAC assays measured by dPCR in comparison with quantitative PCR methods A. Sensitivity of EAC *BCR-ABL1* assay measured by dPCR. *BCR-ABL1* copy numbers measured in 3 independent dilution series prepared from a CML patient with 10% *BCR-ABL1*^IS^. The line indicates the detection limit (0.01% *BCR-ABL1*^IS^) after adjustment to LOD (3.3 copies/sample) calculated from the background amplification in *BCR-ABL1* negative controls – NC. B. Comparison of *BCR-ABL1* ratios (%) measured in K562 ​cell line dilution series using RT-qPCR (black circles) and dPCR (white triangles). Asterisks indicate the significance level determined by Mann-Whitney test: ∗∗∗P<0.0001; ∗∗P<0.001, ∗P<0.05. C. Correlation of *BCR-ABL1* ratios % measured by GeneXpert and dPCR in 36 clinical samples (MR3.0-MR4.5). Pearson correlation analysis was used to determine R^2^= 0.4925 with 95% CI= 0.4851 to 0.8373. D. 3-way comparison of *BCR-ABL1* ratios (%) measured by RT-qPCR (black circles), GeneXpert (black diamond) and dPCR (white triangles) in 12 samples from CML patients in MR3.0-MR4.5. The significance between the methods was determined by Wilcoxon test: RT-qPCR vs. GeneXpert P∗∗=0.0049; dPCR vs GeneXpert P∗∗∗=0.0010.

To further assess the sensitivity of dPCR, we performed quantification of *BCR-ABL1* in 3 independent dilution series. We used a cDNA sample from a CML patient with 10% *BCR-ABL1*^IS^, which was serially diluted into *BCR ABL1* negative control. Digital PCR detected *BCR-ABL1* transcript all sample dilutions down to 0.0001% *BCR-ABL1*^IS^, however after correction to LOD (3.3 copies/sample), the final sensitivity was corresponding to MR4.0 (0.01% *BCR-ABL1*^IS^) (Fig. 1A, Supplementary Table 3).

### 3.2 Comparison of RT-qPCR and dPCR quantification

For comparison of quantitative and digital PCR, we used K562 ​cell line dilution series, corresponding to 10%, 1%, 0.1%, 0.01%, and 0.0032% *BCR-ABL1*^IS^. Despite overall correlation of ratios (Supplementary Fig. 2), we observed significant differences in copy numbers quantification between RT-qPCR and dPCR. In detail, in the samples containing high transcript levels (10%–0.1% *BCR-ABL1*^IS^), RT-qPCR detected significantly more *BCR-ABL1* copies than dPCR (P ​< ​0.0001). Conversely, in the sample with low transcript levels (0.0032% *BCR-ABL1*^IS^), RT-qPCR quantified significantly less *BCR-ABL1* copies compared to dPCR (P ​= ​0.0477). Moreover, in all sample categories dPCR detected significantly less *ABL1* copies (P ​< ​0.0001) (Supplementary Fig. 3; Supplementary Table 4). As a result, *BCR-ABL1*% ratios assessed by RT-qPCR were underestimated, even after correction to IS, compared to dPCR ratios (Fig. 1B; Supplementary Table 5).

The discrepancy of copy numbers was also observed in samples from patients in major and deep molecular response (≤MR3) (n ​= ​12). RT-qPCR still quantified significantly more *ABL1* copies than dPCR in these samples (median copies of 133,971 by RT-qPCR vs. 73,333 by dPCR) (Supplementary Table 6, Supplementary Fig. 4). The *BCR-ABL1* copy numbers showed similar trend.

### 3.3 *BCR-ABL1* monitoring in clinical CML samples

A total of 44 CML patients, routinely monitored by GeneXpert, were tested by dPCR. Patient samples were categorized according to the *BCR-ABL1*^IS^ transcript level as follows: ≤10% (n ​= ​3), ≤1% (n ​= ​3), ≤0.1% (n ​= ​14), ≤0.01% (n ​= ​11) and ≤0.0032% (n ​= ​13). We observed significant differences in the ratios measured by dPCR and GeneXpert in the patients with low transcript levels (n=36; ≤0.1% *BCR-ABL1*^IS^) (Supplemental Figs. 5 and 6), which also resulted in low correlation between the methods (R^2^=0.49; Fig. 1C). Overall, 56% (25/44) of CML patients were placed into different MR categories by dPCR.

In addition, in 12/44 patients (≤0.1% *BCR-ABL1*^IS^) 3-way comparison of *BCR-ABL1* rations was performed as shown in Fig. 1D. Both ratios generated by RT-qPCR and dPCR were significantly different from GeneXpert ratios (P_RT-qPCR_ ​= ​0.0049; P_dPCR_ ​= ​0.0010).

Finally, in 40% (10/25) of CML patients, evaluated by GeneXpert as *BCR-ABL1* negative, dPCR was able to detect *BCR-ABL1* transcript (Supplementary Table 7). However, only 8% of these patients (2/25) were evaluated as positive, with *BCR-ABL1* concentration above LOD (3.3 *BCR-ABL1* copies).

## 4 Discussion

The sensitivity of dPCR for *BCR-ABL1* quantification has already been demonstrated, showing its superiority compared to RT-qPCR (LOD up to MR5.5) [12]. In our study, we showed that dPCR can attain sensitivity above MR5.0. However, we also observed a false positive amplification in *BCR-ABL1* negative controls, not distinguishable from the lowest copy numbers in positive samples. Therefore, final sensitivity was decreased to MR4.0.

The assessment of the false positivity and the detection limit is especially important for the assays, monitoring minimal residual disease. The EAC assays for *BCR-ABL1* detection have been validated on dPCR in the studies of Franke and Maier [9,10], revealing up to 5% false positivity. In our study, we observed a fairly high FPR of 6%. Alikian [7] described similarly increased false positivity in a study comparing three dPCR platforms, using the same combination of an EAC qPCR assays in multiplex, suggesting that this combination and type of assays might not be ideal for dPCR [7,8,13]. Indeed, Andersen and Pallisgaard reported that using a combination of *GUS* and *BCR* control gene assays, or *GUS* alone, resulted in sensitivity of MR5.5 [14]. Most recently, Maier’s group validated new multiplex *BCR-ABL1* and *ABL1* dPCR assays with low-background, 98% specificity, and sensitivity of MR5.0 [10].

Herein, the *BCR-ABL1* and *ABL1* copy numbers quantified in K562 ​cell line and in CML patients by dPCR significantly differed from those detected by RT-qPCR. Franke et al. obtained similar results for samples with ≤0.1 BCR*-ABL1*% measured by dPCR, with significant redistribution of the patients into MR categories relative to RT-qPCR [8]. In addition, similar to our observation, Folta reported the discrepancy in *ABL1* copies measured by two methods (median copies of 180,511 by RT-qPCR vs. 39,960 by dPCR) [15]. Since the cDNA synthesis was performed using the same RT enzyme, we hypothesize that differences among the two methods are likely caused by quantification principles (plasmid standard versus absolute quantification). In addition, dPCR outperformed RT-qPCR in quantifying the lowest transcript levels, which are most likely at the lower detection limit of RT-qPCR. We observed analogous observations when comparing dPCR with GeneXpert. In clinical samples with ≤0.1 BCR*-ABL1*% and *BCR-ABL1* negative samples dPCR yielded more sensitive measurements, GeneXpert assay used for this comparison was validated to MR4.0 [16], which might influence the discrepancy at the lowest transcript levels (<0.01% *BCR-ABL1*^IS^). Still, more than half of the patients in MR3.0 - MR4.0 were categorized into different MR classes by dPCR. Similarly, Folta reported consistent MR levels only in 29/50 samples in a comparison of dPCR with more sensitive GeneXpert Ultra kit with sensitivity of MR4.5. [15]

Our study demonstrated that droplet dPCR, tested with standard EAC assays, provided a detection limit of above 3 BCR*-ABL1* copies/sample, which corresponded to sensitivity of conventional quantitative methods. Nevertheless, dPCR categorized more than 50% of the CML patients into different MR categories compared to quantitative GeneXpert. CML patients in deep molecular response considered for TKI cessation could benefit from more sensitive monitoring using well optimized dPCR assays, however an international standardization independent from RT-qPCR would be required to promote dPCR as an alternative method.

## CRediT authorship contribution statement

**Dagmar Smitalova:** Investigation, Writing – original draft, preparation. **Dana Dvorakova:** Resources, Conceptualization. **Zdenek Racil:** Resources, Writing – review & editing. **Marianna Romzova:** Conceptualization, Methodology, Supervision.

## Declaration of competing interest

Authors DS, DD, ZR, and MR have no competing interests.
