# Supplementary material for: Digital PCR can provide improved BCR-ABL1 detection in chronic myeloid leukemia patients in deep molecular response and sensitivity of standard quantitative methods using EAC assays
Source: Pract Lab Med. 2021 Mar 9;25:e00210. doi: 10.1016/j.plabm.2021.e00210 (PMC7985703; doi:10.1016/j.plabm.2021.e00210)
Supplement: Multimedia component 1 [file mmc1.docx]

**SUPPLEMENTARY DATA**

**Digital PCR can provide improved BCR-ABL1 detection in chronic myeloid leukemia patients in deep molecular response and sensitivity of standard quantitative methods using EAC assays.**

*Dagmar Smitalova,^1,2^ Dana Dvorakova,^3^ Zdenek Racil,^4,5^ and Marianna Romzova^1^*

^1^Department of Molecular Medicine, Central European Institute of Technology, Masaryk University, Brno, Czech Republic

^2^Department of Internal Medicine, Hematology and Oncology, Faculty of Medicine, Masaryk University, Brno, Czech Republic

^3^Centre of Molecular Biology and Gene Therapy, University Hospital Brno, Czech Republic

^4^Internal Haematology and Oncology Clinic, University Hospital Brno, Czech Republic

^5^Institute of Hematology and Blood Transfusion, Prague, Czech Republic

Contact: [romzova.m@gmail.com](mailto:romzova.m@gmail.com)

LIST OF SUPPLEMENTARY TABLES

**Supplementary Table 1**: Characteristics of CML patients.

**Supplementary Table 2**: Performance of BCR-ABL1 assay measured on dPCR.

**Supplementary Table 3**: BCR-ABL1 % ratios measured by dPCR in 3 independent dilution series prepared from CML patient sample.

**Supplementary Table 4**: Comparison of analytical performance of dPCR and RT-qPCR using K562 cell line dilution series.

**Supplementary Table 5**: Comparison of BCR-ABL1 ratios measured by dPCR and RT-qPCR in K562 cell line dilution series.

**Supplementary Table 6**: Comparison of BCR-ABL1 ratios measured by dPCR and GeneXpert in selected patients in deep molecular response.

**Supplementary Table 7**: Correlation dPCR and GeneXpert quantification in BCR-ABL1 negative CML patients.

LIST OF SUPPLEMENTARY FIGURES

**Supplementary Figure 1**: Evaluation of EAC BCR-ABL1 assay linearity on dPCR using an ERM AD623 calibrator.

**Supplementary Figure 2:** Correlation of BCR-ABL1 transcript levels in K562 cell line samples obtained by RT-qPCR and dPCR.

**Supplementary Figure 3:** Comparison of BCR-ABL1 and ABL1 quantification in K562 cell line using RT-qPCR and dPCR.

**Supplementary Figure 4:** Comparison of BCR-ABL1 and ABL1 copy numbers measured by RT-qPCR and dPCR clinical samples.

**Supplementary Figure 5**: Comparison of dPCR and GeneXpert BCR-ABL1 ratios (%) measured in clinical samples.

**Supplementary Figure 6**: Paired analysis of dPCR and GeneXpert BCR-ABL1 ratios (%) in MR groups.

SUPPLEMENTAL METHODS

**Supplementary Table X1**: List of tested EAC primers and probes.

TABLES

**Supplementary Table 1**: Characteristics of CML patients.

| **No. of patients/samples** | 66/70 |
| --- | --- |
| **Gender (Female/Male)** | 34/32 |
| **Age at the time of sample collection** |  |
| Median (range) | 65 (31 - 82) |
| **Patient classification by qPCR (% *BCR-ABL1*^IS^)** | |
| 10% | 3 |
| 1% | 3 |
| MR3.0 | 13 |
| MR4.0 | 11 |
| MR4.5 | 14 |
| Undetectable transcript levels | 25 |

*_IS – international scale_*

**Supplementary Table 2**: Performance of *BCR-ABL1* assay measured on dPCR.

| **Negative controls** | **Number of samples** | **FPR** | **LOB (cp/sample)** | **LOB (droplets)** | **LOD (cp/sample)** |
| --- | --- | --- | --- | --- | --- |
| NTC | 28 | 4% | 1.4 | 1 | 1.5 |
| BCR-ABL1 neg | 52 | 6% | 3.2 | 2 | 3.3 |

*_NTC – no template control;_* ***_BCR-ABL1 neg - BCR-ABL1 negative controls_****_; FPR – false positivity rate, LOB – limit of blank; LOD – limit of detection; cp – copies._*

**Supplementary Table 3**: *BCR-ABL1* % ratios measured by dPCR in 3 independent dilution series prepared from CML patient sample.

| ***BCR-ABL1* dilution series** | | **1st analysis** | **2nd analysis** | **3rd analysis** | **AVERAGE** |
| --- | --- | --- | --- | --- | --- |
| 10% | log1 | NA | 6.2929 | 23.463 | 9.9187 |
| 1% | log2 | 1.3612 | 0.5364 | 3.5142 | 1.8039 |
| 0.1% | log3 | 0.1541 | 0.0513 | 0.3583 | 0.1879 |
| 0.01% | log4 | 0.0125 | 0.0052 | 0.0342 | 0.0173 |
| 0.001% | log5 | 0.0000 | 0.0000 | 0.0037 | 0.0012 |
| 0.0001% | log6 | 0.0000 | 0.0009 | 0.0020 | 0.0010 |

*_NA- not measured._*

**Supplementary Table 4**: Comparison of analytical performance of dPCR and RT-qPCR using K562 cell line dilution series.

|  |  | **N** |  | **Mean CN** | |  | **SD** |  | **CV%** |  |
| --- | --- | --- | --- | --- | --- | --- | --- | --- | --- | --- |
| **Sample category** | **Transcript** | **RT-qPCR** | **dPCR** | **RT-qPCR** | **dPCR** | | **RT-qPCR** | **dPCR** | **RT-qPCR** | **dPCR** |
| 10% | *ABL1* | 18 | 18 | 188 702 | 78 454 | | 26 547 | 14 355 | 14 | 18 |
| 1% | *ABL1* | 18 | 18 | 153 143 | 76 494 | | 25 153 | 13 295 | 16 | 17 |
| 0,1% | *ABL1* | 18 | 18 | 154 790 | 69 259 | | 16 714 | 6 605 | 11 | 10 |
| 0,01% | *ABL1* | 18 | 18 | 137 224 | 77 318 | | 23 413 | 10 593 | 17 | 14 |
| 0.0032% | *ABL1* | 18 | 18 | 151 011 | 82 876 | | 19 320 | 3 911 | 13 | 5 |
| 10% | *BCR-ABL1* | 18 | 17 | 32 724 | 19 316 | | 3 133 | 2 449 | 10 | 13 |
| 1% | *BCR-ABL1* | 18 | 18 | 2 603 | 1 747 | | 498 | 242 | 19 | 14 |
| 0,1% | *BCR-ABL1* | 18 | 17 | 219 | 143 | | 35 | 25 | 16 | 17 |
| 0,01% | *BCR-ABL1* | 18 | 17 | 22 | 23 | | 8 | 9 | 35 | 37 |
| 0.0032% | *BCR-ABL1* | 18 | 16 | 6 | 8 | | 3 | 4 | 60 | 51 |

*_N – number of, CN – copy numbers, SD – standard deviation, CV – coefficient of variance._*

**Supplementary Table 5**: Comparison of BCR-ABL1 ratios measured by dPCR and RT-qPCR in K562 cell line dilution series.

|  | | | **Mean ratio (%)** | | | | **SD** | | |  | **CV%** | | |
| --- | --- | --- | --- | --- | --- | --- | --- | --- | --- | --- | --- | --- | --- |
| **Sample category** | **N** | **RT-qPCR** | | **RT-qPCR IS** | **dPCR** | **RT-qPCR** | | **RT-qPCR IS** | **dPCR** | **RT-qPCR** | | **RT-qPCR IS** | **dPCR** |
| 10% | 6 | 17.4 | | 10.8 | 24.5 | 1.5 | | 0.9 | 3.8 | 8 | | 9 | 16 |
| 1% | 6 | 1.7 | | 1.1 | 2.3 | 0.2 | | 0.1 | 0.3 | 11 | | 11 | 15 |
| 0,1% | 6 | 0.1 | | 0.1 | 0.2 | 0.02 | | 0.01 | 0.03 | 16 | | 16 | 14 |
| 0,01% | 6 | 0.02 | | 0.01 | 0.03 | 0.004 | | 0.003 | 0.012 | 27 | | 28 | 39 |
| 0.0032% | 6 | 0.004 | | 0.002 | 0.009 | 0.002 | | 0.001 | 0.003 | 51 | | 72 | 35 |

*_N – number of, CN – copy numbers, SD – standard deviation, CV – coefficient of variance, IS – international scale._***Supplementary Table 6**: Comparison of BCR-ABL1 ratios measured by dPCR and GeneXpert in selected patients in deep molecular response.

|  | ***ABL1* (mean CN)** | | ***BCR-ABL1* (mean CN)** | | **Ratios** | | |
| --- | --- | --- | --- | --- | --- | --- | --- |
| **Patient** | **RT-qPCR** | **dPCR** | **RT-qPCR** | **dPCR** | **RT-qPCR (IS)** | **dPCR** | **GeneXpert (IS)** |
| **12** | 134166 | 75467 | 208 | 101 | 0,1547 | 0,1343 | 0,047 |
| **14** | 115653 | 59840 | 66 | 22 | 0,0567 | 0,0372 | 0,033 |
| **22** | 152624 | 78300 | 113 | 46 | 0,0741 | 0,0587 | 0,012 |
| **29** | 110467 | 67933 | 7 | 6 | 0,0065 | 0,0083 | 0,005 |
| **31** | 178941 | 95800 | 29 | 16 | 0,0161 | 0,0169 | 0,004 |
| **32** | 104343 | 59353 | 8 | 6 | 0,0077 | 0,0095 | 0,003 |
| **33** | 61311 | 34913 | 0 | 1 | 0,0000 | 0,0025 | 0,003 |
| **35** | 144680 | 83400 | 6 | 3 | 0,0039 | 0,0038 | 0,002 |
| **41** | 158803 | 76267 | 11 | 4 | 0,0067 | 0,0052 | 0,001 |
| **42** | 109568 | 68333 | 1 | 2 | 0,0009 | 0,0023 | 0,001 |
| **43** | 182975 | 82200 | 78 | 31 | 0,0428 | 0,0383 | 0,001 |
| **44** | 133777 | 71200 | 19 | 3 | 0,0141 | 0,0038 | 0,001 |
| **Median** | 133972 | 73333 | 15 | 6 | 0,0109 | 0,0089 | 0,0030 |

*_CN – copy numbers, IS – international scale._*

**Supplementary Table 7**: Comparison of dPCR and GeneXpert quantification in BCR-ABL1 negative CML patients.

|  | **GeneXpert** | | | | | **dPCR** | | |
| --- | --- | --- | --- | --- | --- | --- | --- | --- |
|  | **BCR-ABL1 ratio % (IS)** | | | | **ABL1 (copies/sample)** | | **BCR-ABL1 (copies/sample)** | **BCR-ABL1 ratio %** |
| **Patient #** | **S1** | **S2** | **S3** | **S4** | **S4** | | | |
| 1 | 0.001 | 0.001 | 0 | 0 | 101 146 | | 0 | 0 |
| 2 | 0.003 | 0 | 0 | 0 | 91 421 | | 0 | 0 |
| 3 | 0.002 | 0 | 0 | 0 | 121 558 | | 0 | 0 |
| 4 | 0 | 0 | 0 | 0 | 149 153 | | 1.52 | 0.001 |
| 5 | 0 | 0 | 0 | 0 | 156 948 | | 0 | 0 |
| 6 | 0 | 0 | 0 | 0 | 85 814 | | 0 | 0 |
| 7 | 0 | 0 | 0 | 0 | 142 206 | | 1.3 | 0.001 |
| 8 | 0 | 0 | 0 | 0 | 121 610 | | 0 | 0 |
| 9 | 0.001 | 0 | 0 | 0 | 120 909 | | 0 | 0 |
| 10 | 0 | 0 | 0 | 0 | 85 948 | | 0 | 0 |
| 11 | 0 | 0 | 0 | 0 | 93 595 | | 0 | 0 |
| 12 | 0 | 0 | 0 | 0 | 139 163 | | 1.26 | 0.001 |
| 13 | 0 | 0 | 0 | 0 | 111 362 | | 1.31 | 0.001 |
| 14 | 0 | 0 | 0 | 0 | 112 422 | | 0 | 0 |
| 15 | 0 | 0 | 0.002 | 0 | 161 340 | | 0 | 0 |
| 16 | 0.065 | 0.14 | 0.026 | 0 | 230 800 | | 120 | 0.052 |
| 17 | 0.001 | 0 | 0 | 0 | 210 400 | | 1.6 | 0.001 |
| 18 | 0 | 0.003 | 0 | 0 | 218 600 | | 0 | 0 |
| 19 | 0.001 | 0.001 | 0.003 | 0 | 175 620 | | 7 | 0.004 |
| 20 | 0.001 | 0.002 | 0.001 | 0 | 250 400 | | 0 | 0 |
| 21 | 0 | 0.001 | 0 | 0 | 188 740 | | 1.4 | 0.001 |
| 22 | 0 | 0 | 0.001 | 0 | 218 800 | | 1.4 | 0.001 |
| 23 | 0 | 0 | 0 | 0 | 225 000 | | 0 | 0 |
| 24 | 0.001 | 0.001 | 0 | 0 | 191 760 | | 1.4 | 0.001 |
| 25 | 0 | 0 | 0 | 0 | 178 000 | | 0 | 0 |

*_#- number. S1 – S3 – sampling during follow-up (3 months apart) performed by GeneXpert, S4 – sampling when sample was BCR-ABL1 negative by GeneXpert; cp/sample – copies detected in triplicate of each sample_*

**FIGURES**

**Supplementary Figure 1**: Evaluation of EAC *BCR-ABL1* assay linearity on dPCR using an ERM AD623 calibrator.


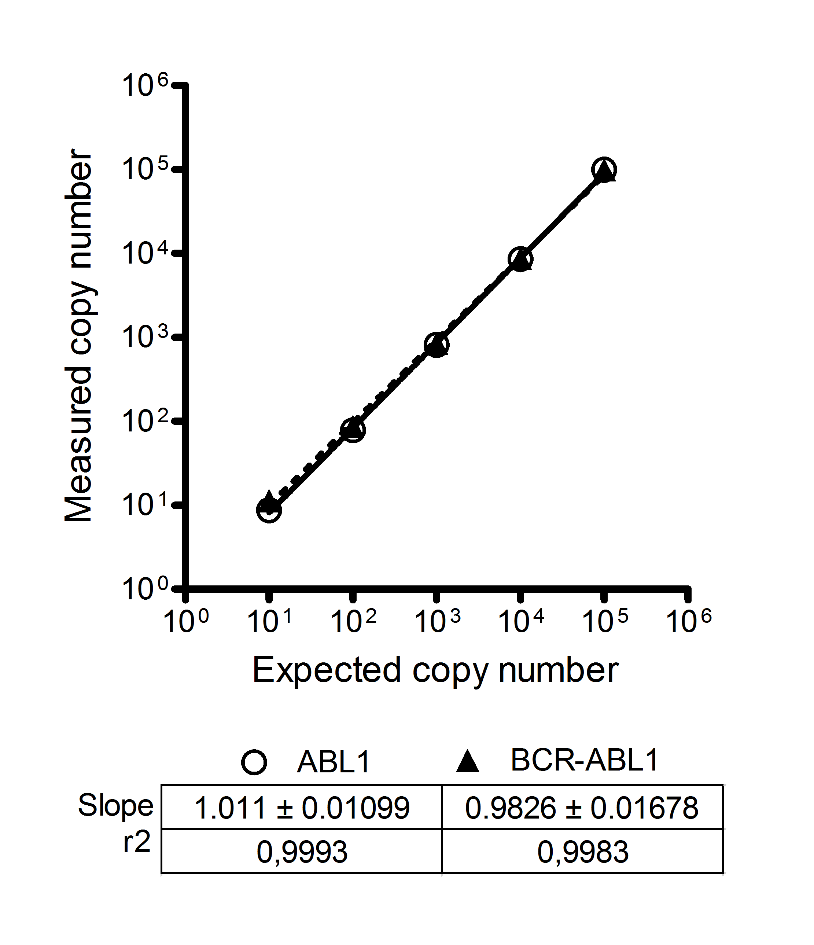


**Supplementary Figure 2:** Correlation of *BCR-ABL1* transcript levels in K562 cell line samples obtained by RT-qPCR and dPCR.


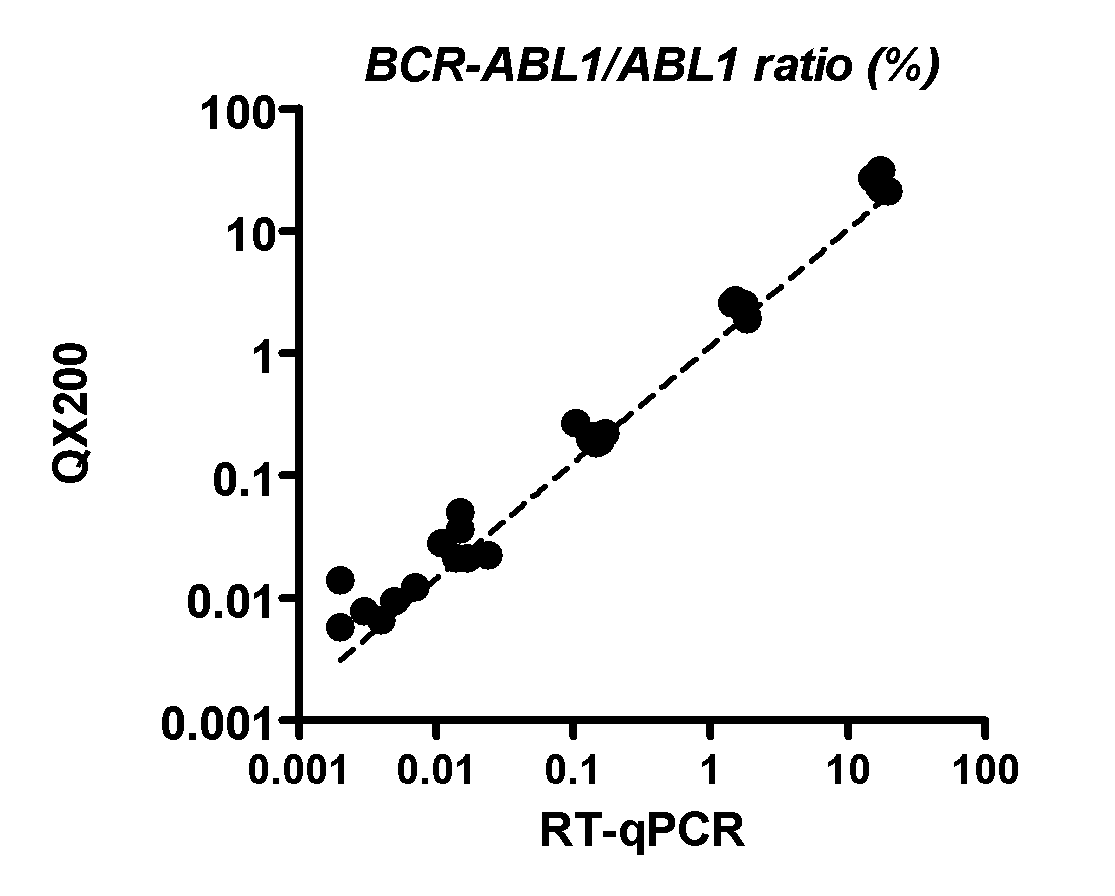


|  | |
| --- | --- |
| Number of XY Pairs | 30 |
| **Pearson r** | **0.9765** |
| 95% confidence interval | 0.9507 to 0.9889 |
| P value (two-tailed) | P<0.0001 |
| P value summary | *** |
| Is the correlation significant? (alpha=0.05) | Yes |
| **R squared** | **0.9536** |

**Supplementary Figure 3:** Comparison of *BCR-ABL1* and *ABL1* quantification in K562 cell line using RT-qPCR and dPCR.


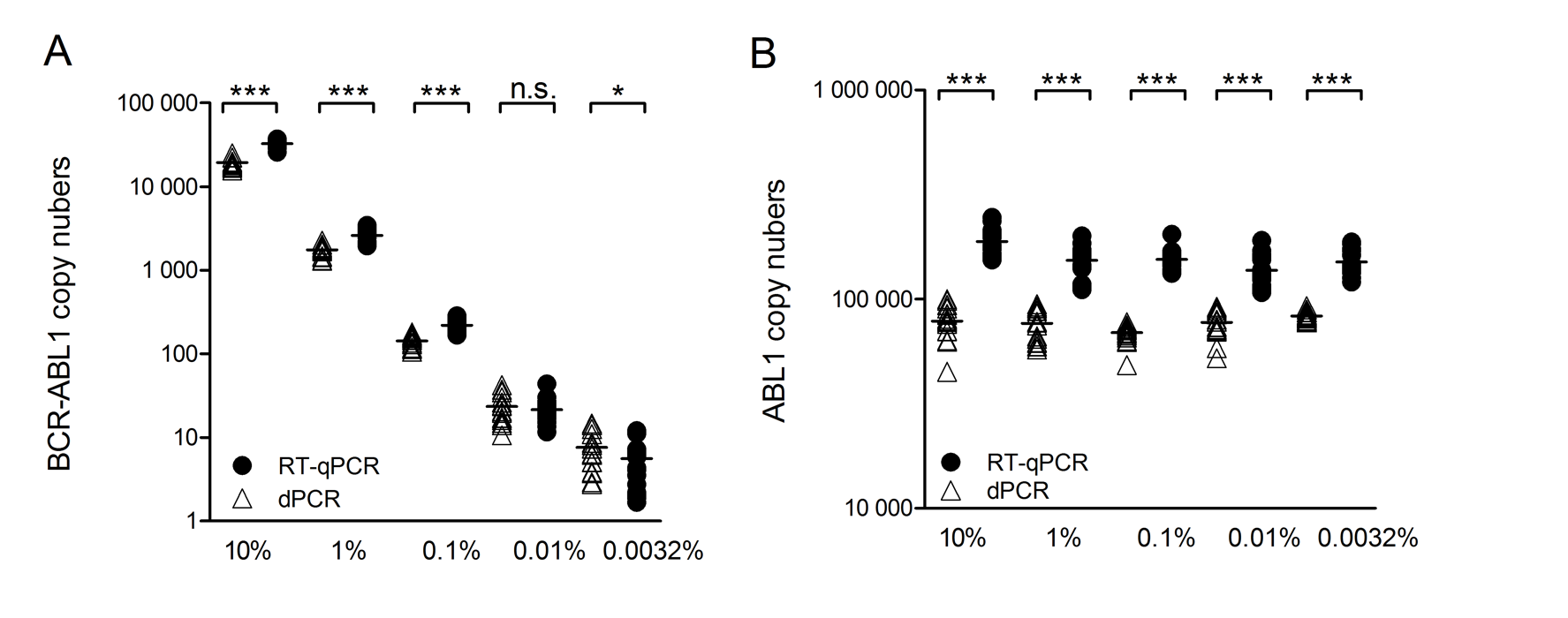


Mean *BCR-ABL1* (left graph) and *ABL1* (right graph) copy numbers (***P<0.001; **P<0.01, *P<0.05) measured by RT-qPCR (black circles) and dPCR (empty triangles). Asterisks are indicating the level of significance (***P<0.001; **P<0.01, *P<0.05) of copy number differences among sample groups: 10% - 0.0032% *BCR-ABL1*^IS^ generated from K562 cell line.

**Supplementary Figure 4:** Comparison of *BCR-ABL1* and *ABL1* copy numbers measured by RT-qPCR and dPCR patient samples.

*BCR-ABL1* (left graph) and *ABL1* (right graph) copy numbers measured by RT-qPCR (black circles) and dPCR (empty triangles). Asterisks are indicating the level of significance (***P<0.001; **P<0.01) of copy number differences among methods.

**Supplementary Figure 5**: Comparison of dPCR and GeneXpert *BCR-ABL1* ratios (%) measured in clinical samples.

Red triangles indicate patient samples categorized into different MR group by dPCR in comparison with GeneXpert, while white triangles indicate patients categorized into same groups.

**Supplementary Figure 6**: Paired analysis of dPCR and GeneXpert *BCR-ABL1* ratios (%) in MR groups.

Asterisks are indicating the level of significance (****P<0.0001; **P<0.01) of ratio differences between methods in patient samples grouped according to their MR level.

**SUPPLEMENTARY METHODS**

**K562 cell culture, sample processing and storage**

K562 cell line was cultured in RPMI medium supplemented with 10% FBS and 1% (v/v) penicillin/streptomycin. For RT-qPCR and dPCR testing, peripheral blood from CML patients and *BCR-ABL1* negative controls (healthy controls) was subjected to red blood cell lysis. Using 600 µL of Qiagen RLT buffer, a lysate was prepared from total leukocytes and K562 cells. RNA was extracted from the lysate using Qiagen RNeasy Mini Kit according to the manufacturer recommendations, including on-column DNase digestion.

For the GeneXpert testing of patient samples, 200 µL of fresh peripheral blood (PB) was used as an input material and processed using GeneXpert cartridges according to the manufacturer’s protocol. Additional 10 ml of fresh PB were subjected to red blood cell lysis and total leukocytes were lysed in 600 µL of Qiagen RLT buffer. For parallel dPCR evaluation, RNA was extracted from RLT lysates.

Freshly isolated RNA was either submitted directly to reverse transcription step or stored on -80.

All experiments, including reverse transcription, digital and RT-qPCR were executed in accordance with the Minimum Information for Publication of Quantitative Real-Time (MIQE) and Digital (dMIQE) PCR Experiments Guidelines [1,2].

**Quantitative reverse transcription PCR**

Complementary DNA (cDNA) was prepared using random hexamer primers with Invitrogen SuperScript II Reverse Transcriptase according to the manufacturer’s instructions. Primers and probes were adapted from the EAC protocol (**Supplementary Table** X**1**)*.* *ABL1* gene was selected as a reference gene for normalization according to EAC recommendations [3,4]. Duplicate qPCR reactions were carried out using Thermo Fisher Scientific ABsolute qPCR ROX Mix with 300 nM of each primer (Generi Biotech) and 200 nM of probe (Generi Biotech). RT-qPCR results were presented as a percentage of *BCR-ABL1* to *ABL1* ratio. Laboratory/assay-specific conversion factors (CF), determined by comparison to an IS reference assay, were used to convert our local results to International Scale (IS). The RT-qPCR test was validated by European Treatment Outcome Study (EUTOS) [5] at MR4.5.

**Digital PCR**

Complementary DNA was prepared from a total RNA using SuperScript II Reverse Transcriptase for comparison of dPCR with RT-qPCR in K562 cell line samples, and Invitrogen SuperScript VILO Master Mix for analysis of FPR, LOB, LOD and sensitivity in *BCR-ABL1* negative controls and patient samples, and for comparison between dPCR, RT-qPCR and GeneXpert in patient samples. The same EAC qPCR assays as in conventional RT-qPCR were used. To establish the optimal dPCR workflow, a primer/probe concentration testing and gradient dPCR were performed prior to the measurement of the cell line and clinical samples (data not shown).

Triplicate qPCR reactions were carried out with 450 nM of each primer (Generi Biotech) and 125nM of each probe (Generi Biotech) using Bio-Rad ddPCR Supermix for Probes (no dUTP), as described previously [6,7]. The results (copies per microliter multiplied by reaction volume; cp/sample) were generated by QuantaSoft Analysis Pro v.1.0 program.

**Statistical analysis – FPR, LOB, LOD**

The FPR was calculated as a percentage of false positive samples (N _pos_) from all blank samples (N _total_) according to the following formula: FPR = N _pos_/N _total_ *100 (%).

Limit of blank (LOB) was calculated as: LOB = Mean _blank_ + 1.645*SD _blank_. Where Mean _blank_ equals the average *BCR-ABL1* concentration in all positive blank samples (in copies/sample) and SD _blank_ is the standard deviation of *BCR-ABL1* copies/sample in positive blank samples. Limit of detection (LOD) was calculated as: LOD = LOB + 1.645*SDχ. Where χ represents a low concentration sample; in this study we used samples having the lowest *BCR-ABL1* concentration in the dilution series.

**Supplementary Table X1**: List of tested EAC primers and probes.

| **Transcript** | **Accession number** | **Amplicon size** | **Oligo type** | **Location** | **Sequence (5'->3') (modification)** | **Oligo length** | **Probe modification** | |
| --- | --- | --- | --- | --- | --- | --- | --- | --- |
|  |  |  |  |  |  |  | **5'** | **3'** |
| ***BCR-ABL1*** | AJ131466 (e14a2) | 149 bp (e14a2) | Forward primer | *BCR*  Exon 13 | TCCGCTGACCATCAA**T(Y)**AAGGA | 21 |  |  |
|  | AJ131467 (e13a2) | 74 bp (e13a2) | Reverse primer | *ABL1*  Exon 2 | CACTCAGACCCTGAGGCTCAA | 21 |  |  |
|  |  |  | Probe |  | CCCTTCAGCGGCCAGTAGCATCTGA | 25 | 6-FAM | BHQ |
| ***ABL1*** | NM_005157 (*ABL1* mRNA-a) | 124 bp | Forward primer | *ABL1*  Exon 2-3 | TGGAGATAACACTCTAAGCATAACTAAAGGT | 31 |  |  |
|  | NM_007313 (*ABL1* mRNA-b) |  | Reverse primer | *ABL1*  Exon 3 | GATGTAGTTGCTTGGGACCCA | 21 |  |  |
|  |  |  | Probe |  | CCATTTTTGGTTTGGGCTTCACACCATT | 28 | 6-FAM | BHQ |

**References:**

[1] S.A. Bustin, V. Benes, J.A. Garson, J. Hellemans, J. Huggett, M. Kubista, R. Mueller, T. Nolan, M.W. Pfaffl, G.L. Shipley, J. Vandesompele, C.T. Wittwer, The MIQE guidelines: minimum information for publication of quantitative real-time PCR experiments., Clin. Chem. 55 (2009) 611–22. https://doi.org/10.1373/clinchem.2008.112797.

[2] J.F. Huggett, C.A. Foy, V. Benes, K. Emslie, J.A. Garson, R. Haynes, J. Hellemans, M. Kubista, R.D. Mueller, T. Nolan, M.W. Pfaffl, G.L. Shipley, J. Vandesompele, C.T. Wittwer, S.A. Bustin, The digital MIQE guidelines: minimum information for publication of quantitative digital PCR experiments, Clin. Chem. 59 (2004) 892–902. https://doi.org/10.1373/clinchem.2004.035469.

[3] J. Gabert, E. Beillard, V.H.J. van der Velden, W. Bi, D. Grimwade, N. Pallisgaard, G. Barbany, G. Cazzaniga, J.M. Cayuela, H. Cavé, F. Pane, J.L.E. Aerts, D. De Micheli, X. Thirion, V. Pradel, M. González, S. Viehmann, M. Malec, G. Saglio, J.J.M. van Dongen, Standardization and quality control studies of ‘real-time’ quantitative reverse transcriptase polymerase chain reaction of fusion gene transcripts for residual disease detection in leukemia – A Europe Against Cancer Program, Leukemia. 17 (2003) 2318–2357. https://doi.org/10.1038/sj.leu.2403135.

[4] E. Beillard, N. Pallisgaard, V.H.J. van der Velden, W. Bi, R. Dee, E. van der Schoot, E. Delabesse, E. Macintyre, E. Gottardi, G. Saglio, F. Watzinger, T. Lion, J.J.M. van Dongen, P. Hokland, J. Gabert, Evaluation of candidate control genes for diagnosis and residual disease detection in leukemic patients using “real-time” quantitative reverse-transcriptase polymerase chain reaction (RQ-PCR) - A Europe against cancer program, Leukemia. 17 (2003) 2474–2486. https://doi.org/10.1038/sj.leu.2403136.

[5] M. Baccarani, B. Simonsson, D. Lindörfer, G. Rosti, A.M. Almeida, A. Bogdanovic, R.E. Clark, A. Colita, P.A. Costeas, L. Griskevicius, J. Guilhot, A. Hellmann, K. Indrak, E. Laane, B. Labar, T. Masszi, S. Lejniece, J. Mayer, G. Ossenkoppele, P. Panayiotidis, K. Porkka, S. Saussele, A. Hochhaus, J.L. Steegmann, J. Thaler, A. Turkina, G. Verhoef, A. Zaritskey, I.P. Zupan, F. Rancati, L. Montrucchio, R. Hehlmann, J. Hasford, The European Treatment and Outcome Study (EUTOS) for Chronic Myeloid Leukemia (CML). A Prospective, Population-Based European Registry., Blood. 114 (2009) 4272–4272. https://doi.org/10.1182/blood.V114.22.4272.4272.

[6] L.J. Jennings, D. George, J. Czech, M. Yu, L. Joseph, Detection and quantification of BCR-ABL1 fusion transcripts by droplet digital PCR, J. Mol. Diagn. 16 (2014) 174–179. https://doi.org/10.1016/j.jmoldx.2013.10.007.

[7] M. Alikian, A.S. Whale, S. Akiki, K. Piechocki, C. Torrado, T. Myint, S. Cowen, M. Griffiths, A.G. Reid, J. Apperley, H. White, J.F. Huggett, L. Foroni, RT-qPCR and RT-digital PCR: A comparison of different platforms for the evaluation of residual disease in chronic myeloid leukemia, Clin. Chem. 63 (2017) 525–531. https://doi.org/10.1373/clinchem.2016.262824.
